# Ameliorated biomechanical properties of carotid arteries by puerarin in spontaneously hypertensive rats

**DOI:** 10.1186/s12906-021-03345-8

**Published:** 2021-06-22

**Authors:** Xiaoxia Fang, Sheng Dong, Yun Wu, Yun He, Min Lu, Dandan Shi, Na Feng, Songhe Yin, Yan Jiang, Anhua Zhang, Yan Ding, Qiufang Zhang, Junming Tang, Wenjun Zhang, Xiju He

**Affiliations:** 1 Department of Neurology, Taihe Hospital, Hubei University of Medicine, Shiyan, 442000 China; 2 Department of Anatomy, Hubei University of Medicine, Shiyan, 442000 China; 3 Department of Ultrasound, Taihe Hospital, Hubei University of Medicine, Shiyan, 442000 China; 4 Hubei Key Laboratory of Embryonic Stem Cell Research, Hubei University of Medicine, Shiyan, 442000 China

**Keywords:** Hypertension, Puerarin, TRPC, Vascular remodeling, Biomechanical property

## Abstract

**Background:**

An emerging body of evidence indicates that puerarin (PUE) plays an important role in the treatment of angina pectoris, myocardial ischemia-reperfusion injury, hypertension and other cardiovascular diseases, but how PUE affects the vascular remodeling of hypertensive rats has not been reported yet. This study aimed to investigate the effect and mechanism of PUE on carotid arteries of spontaneously hypertensive rats (SHR) to provide the basis for the clinical application of PUE.

**Methods:**

Thirty male SHR and six male Wistar Kyoto rats (WKY) aged 3 months were used in this study, SHR rats were randomly divided into 5 groups, PUE(40 or 80 mg/kg/d, ip) and telmisartan (TELMI) (30 mg/kg/d, ig) were administrated for 3 months. We use DMT myography pressure-diameter system to investigate biomechanical properties of carotid arteries, 10 μM pan-classical transient receptor potential channels (TRPCs) inhibitor SKF96365, 200 nM specific TRPC6 inhibitor SAR7334 and 100 μM Orai1 inhibitor ANCOA4 were used in the mechanical test.

**Results:**

PUE can significantly decrease systolic and diastolic blood pressure, long-term administration of PUE resulted in a mild reduction of thickness and inner diameter of carotid artery. PUE ameliorate NE-response and vascular remodeling mainly through inhibiting TRPCs channel activities of VSMC.

**Conclusion:**

PUE can ameliorate biomechanical remodeling of carotid arteries through inhibiting TRPCs channel activities of VSMC in spontaneously hypertensive rats.

**Supplementary Information:**

The online version contains supplementary material available at 10.1186/s12906-021-03345-8.

## Introduction

Hypertension is a major cardiovascular risk factor and cause of mortality worldwide [1], blood pressure is regulated by a variety of complex neurohumoral and mechanical signals that together determine systemic vascular tone and resistance [2, 3]. The pathogenesis of hypertension has not yet been completely elucidated; its consensual pathophysiological basis is vascular remodeling. Arterial growth (change in mass) and remodeling (change in structure) in response to altered dynamic mechanical stimuli in hypertension may be more important than previously thought.

Few studies have attempted to quantify in-vivo dynamic mechanical stimuli more broadly as initiators or indicators of arterial disease. Mechanical characterization can be used to test hypertension treatments aimed at reversing mechanical remodeling changes that compromise human health. Large conducting arteries (aorta and carotid artery) in vertebrates are composed of a specialized extracellular matrix designed to provide pulse dampening and reduce the work performed by the heart. Intra-media thickness of carotid artery is a reliable indicator to reflect the changes of vascular function in clinical studies. When the matrix proteins of carotid arteries are altered in hypertension, the arterial wall can be remodeled, the mechanical properties changed, and leading to subsequent cardiac adaptation.

*Pueraria lobata* is widely known as Gegen (Chinese name) in traditional Chinese medicine, puerarin (PUE) is one of the main active ingredients extracted from the root of *P. lobata*. Its chemical name is 8-C-*β*-D-glucopyranosyl-7,4-hydroxy-isoflavone and has a molecular weight of 416.382. PUE plays an important role in the treatment of angina pectoris, myocardial ischemia-reperfusion injury, hypertension and other cardiovascular diseases [4–6]. So far, anti-inflammatory [7], anti-oxidative [8] and anti-arrhythmia [9] effects of PUE have been proven. However, the molecular mechanism underlying the antihypertensive effects of PUE is still not well understood. Recently studies have found that PUE could ameliorate pressure overload-induced cardiac hypertrophy in ovariectomized rats through activation of the PPARα/PGC-1 pathway [10]. The latest research report that PUE exhibit high ACE2-targeting potential, it might be an ideal ponderable drug for COVID-19 through impairing the interaction between S-protein and ACE2 by SPR assays [11], but whether PUE affects the vascular mechanical reconstruction of hypertensive rats has not been reported yet.

The vascular system is a highly dynamic structure that undergoes constant remodeling, physiological as well as maladaptive restructuring processes are associated with altered Ca^2+^ homeostasis in both vascular endothelium and smooth muscle. Studies have reported that calcium deposition on arterial elastic fibers also enhances the wall stiffening [12, 13]. PUE also shows acute inhibitory effects on the functional ion channels, such as Na^+^ channels [14], inward rectified K^+^ channels [15] and LTCC [16] in isolated cardiomyocytes, but the effect of PUE against Ca^2+^ influx still remains controversial [17–19]. Our previous study found that PUE could ameliorate the vasorelaxation of mesenteric artery of SHR rats [20]. In this study, we want to make it clear how PUE affect Ca^2+^ influx and regulate calcium channels of carotid artery in hypertension.

In the present study, we used DMT Myography pressure-diameter system to probe the effect of PUE on carotid remodeling, and conducted [Ca^2+^]i, live-cell fluorescent Ca^2+^ imaging, and choose some calcium channel antagonists to study the effects of PUE on TRPCs channels and vascular tension. We found that PUE can decrease systolic and diastolic blood pressure, long-term administration with PUE resulted in a mild reduction of thickness and inner diameter of carotid artery, PUE can ameliorate NE-response of SHR rats, PUE ameliorate vascular remodeling mainly through inhibiting TRPCs channel activities of vascular smooth muscle cells (VSMC).

## Materials and methods

### Animals and main reagents

Thirty male specific pathogen-free (SPF) SHR and six male WKY rats aged 3 months were purchased from charlers river company, the rats were housed in the SPF Animal Center of Hubei University of Medicine. Animals were kept on a 12 h–12 h light–dark cycle with ad libitum access to food and water. All animal studies were approved by the Animal Care and Utilization Committee of Hubei University of Medicine (approval No. SYXK2016–0031). PUE used for injection (freeze-dried powder, 200 mg/bottle) was provided by Reyang Pharmaceutical Company of China, Telmisartan (TELMI) potassium tablets (100 mg/tablet) were provided by Moshadong Pharmaceutical Company of China. Thirty SHR rats were randomly divided into 5 groups: SHR group (+ Saline); SHR + PUE40 group (PUE 40 mg/kg/d, ip); SHR + PUE80 group (PUE 80 mg/kg/d, ip); SHR + TELMI group (TELMI 30 mg/kg/d, ig). And SHR + PUE + TELMI group (PUE 80 mg/kg, ip + TELMI, 30 mg/kg/d ig). Six WKY rats were taken as the normal control group. Anesthetic procedures were used to ensure that animals not suffer unduly during and after the experimental procedures.

### Blood pressure measurement

Blood pressure of tail arteries were measured at the 0th, 4th, 8th, and 12th week of administration by BP-6 non-invasive blood pressure measuring System (Chengdu Taimeng Technology Company). Blood pressures were needed to be measured three times in a quiet state, systolic blood pressure (SBP) and diastolic blood pressure (DBP) were measured and recorded carefully.

### Ultrasonic test

Ultrasonic test was performed with a Vevo2100 imaging system and a MS250 transducer, which is designed specifically for mice and rats (Visual Sonics). Rats were anesthetized with 2% isoflurane during the ultrasonic test procedure, the diameter and wall thickness of common carotid artery (CCA) were measured during diastole of left ventricle.

### Mechanical test

Pressure myography (DMT 114P, Denmark) is a relatively simple, but sensitive and mechanistically useful technique that can be used to assess the effect of various stimuli on vascular contraction and relaxation, thereby augmenting our insight into the mechanisms underlying cardiovascular disease. Opened the chest to expose the right and left carotid arteries, carefully separated CCA and remove the fat and connective tissue around it, placed CCA in a microfuge tube filled with PSS pre-cooled at 4 °C with 95% O_2_ + 5% CO_2_ ventilation. Then CCA were mounted onto two cannulas connected to a pressurized and sealed system which maintained at constant pressure of 60 mmHg. 10^− 5^ M Norepinephrine (NE) and 10^− 5^ M acetylcholine (Ach) were used to detect vascular activity, NE was used to test smooth muscle contractility and 10^− 5^ M sodium nitroprusside (SNP) to test endothelial-independent smooth muscle relaxation. The lumen and outer diameter of CCA were continuously recorded using a video camera, allowing real time quantification of the vasoconstriction and vasorelaxation respectively, incubated CCA with 10 μM SKF96365 (pan-TRPCs inhibitor), 200 nM SAR7334 (specific TRPC6 inhibitor), or 100 μM ANCOA4 (Orai1 inhibitor) for 30 min, then recorded the outer diameter and pressure changes.

### Histopathological stain

The same location part of carotid arteries of each group were separated and dehydrated, embedded in paraffin, and sliced. HE staining and Masson staining were performed, and the structure changes of CCA were observed under microscope.

### Ca^2+^ imaging experiments

A7R5 cells were incubated with different concentrations of PUE for 24 h before experiment, Fura2-AM was used as the fluorescent indicator for Ca^2+^. For functional studies of cytosolic Ca^2+^, the cells were loaded with 2 μM Fura2-AM for 30 min at room temperature in serum-free medium, After incubation, cells were perfused with Ca^2+^-free HBSS solution of 0.1 μM AngII,Cells were then rapidly switched to 2 mM Ca^2+^- HBSS solution with AngII, Cytosolic Ca^2+^ was monitored with Ca^2+^-imaging system (LAMBDA) with an Olympus IX51 inverted fluorescence microscope and Slidebook software, using excitation wavelengths of 340 and 380 nm to detect Fura-2/Fura2-Ca^2+^ fluorescence emissions at 510 nm.

### Intracellular calcium concentration measurement

Intracellular calcium concentration measurement was conducted using fluorescent dye Fluo-3/AM staining. After treatment, A7R5 cells were re-suspended in Fluo-3/AM working solution (5 μM) for 30 min in the dark and washed twice with PBS. After that, cells were incubated in culture medium for another 20 min in dark. Intracellular Ca^2+^ influx was taken by fluorescence microscope (Leica Microsystems).

### Statistical analysis

The data are presented as means ± SD; Statistical analyses were performed by ANOVA followed by Bonferroni corrections for multiple comparisons between groups. Significance was assumed at *p* < 0.05, All statistical analyses were performed using SPSS 18.0 Software (SPSS Inc., Chicago, IL).

## Results

### PUE significantly decreased SBP and DBP of SHR rats

**Fig. 1:**
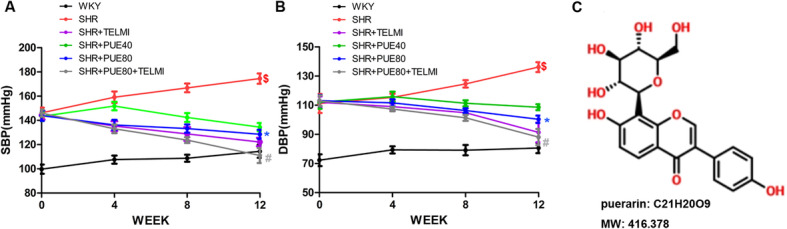
PUE significantly decreased SBP and DBP of SHR rats. **A** SBP of SHR group was significantly higher than WKY group, 80 mg/kg/d PUE could decrease SBP of SHR, systolic blood pressure: SBP; **B** DBP of SHR group was higher than WKY group, 80 mg/kg/d PUE could decrease DBP of SHR; diastolic blood pressure: DBP. **C** The chemical structure of puerarin. $ *p* < 0.05 vs WKY; * *p* < 0.05 vs SHR; # *p* < 0.05 vs SHR + TELMI. (Repeated measures ANOVA)

Blood pressure of rat tail arteries were measured on the 0th, 4th, 8th, and 12th week. As shown in Fig. 1, the results showed that the SBP and DBP of SHR group were significantly higher than WKY group (*p* < 0.05), it illustrated that the high blood pressure of SHR rats were induced successfully at the end of 12 weeks. SBP (Fig. 1A) and DBP (Fig. 1B) of SHR + PUE80 group were lower than those of SHR group (*p* < 0.05). Compared to SHR + TELMI group, the SBP and DBP of the SHR + PUE80 + TELMI group also decreased significantly, it showed that combination of TELMI and PUE could obviously decrease SBP and DBP, which suggested PUE and TELMI had a synergistic effect on decreasing blood pressure (*p* < 0.05).

### PUE obviously decreased thickness and inner diameter of carotid artery

**Fig. 2:**
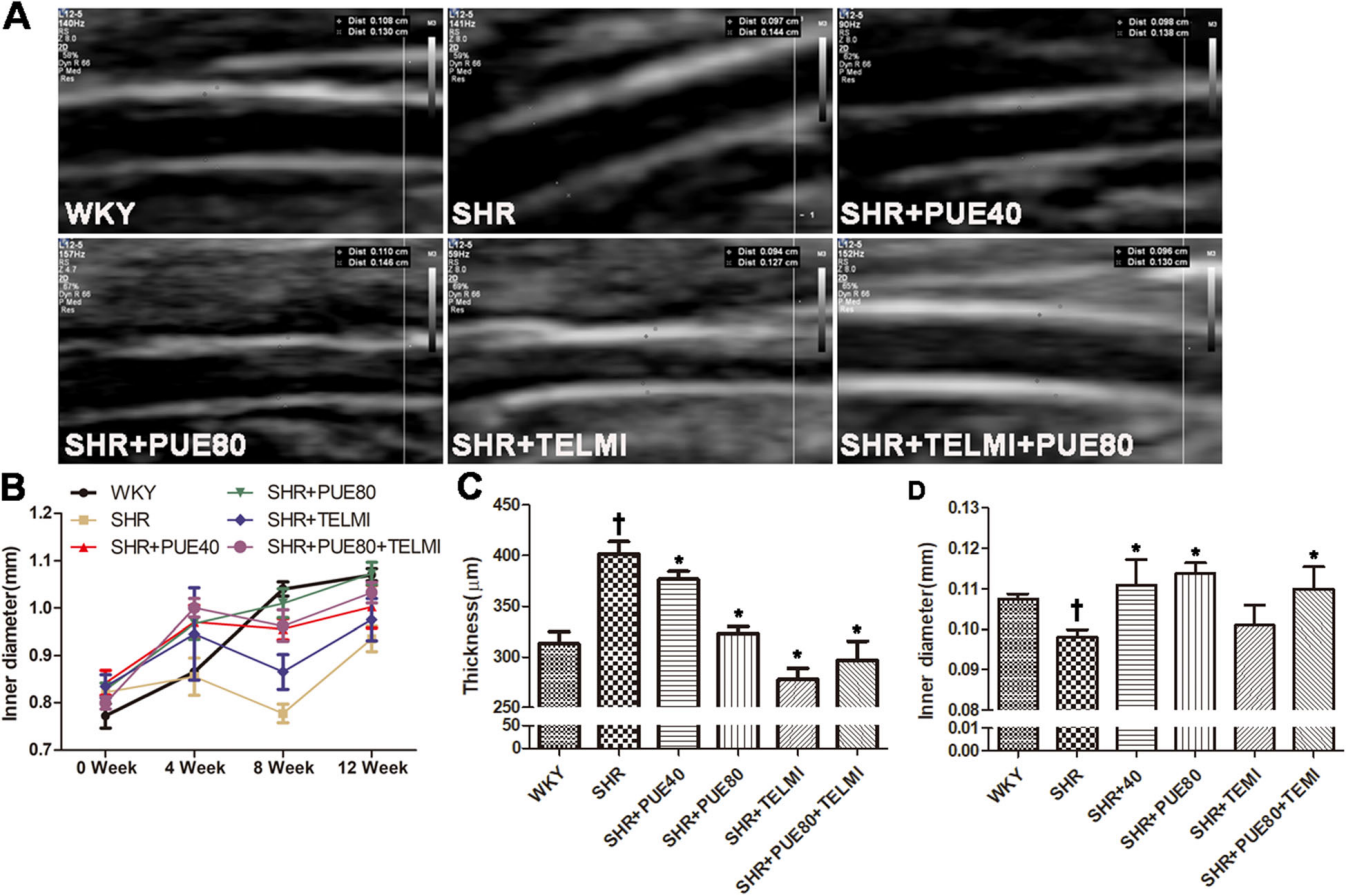
Effect of PUE in common carotid artery with ultrasound image. **A** A representative ultrasound record of CCA at the end of 12 weeks. **B** Inner diameter changes of CCA with PUE during 0–12 weeks. **C** Histogram analysis of thickness changes of CCA with PUE at the end of 12 weeks. **D** Histogram analysis of inner diameter changes of CCA with PUE at the end of 12 weeks. † *p* < 0.05 vs WKY, * *p* < 0.05 vs SHR. (Repeated measures ANOVA)

In order to better probe the pathological process of hypertension and the role of PUE, the diameters of CCA were measured by ultrasound. The ultrasound images of CCA at the 12th week were shown in Fig. 2A. Changes of inner diameter of CCA during 12 weeks were shown in Fig. 2B. The histogram analysis illustrated PUE could obviously decrease the thickness and inner diameter at the 12th week in Fig. 2C and D. It suggested long-term administration of PUE could change CCA structure of SHR rats. Studies have reported that TELMI is a peptide angiotensin II receptor antagonist, it can optionally and irreversibly block ATI receptors used in essential hypertension, that was why the inner diameter and thickness were smaller in TELMI-administrated groups.

### PUE ameliorated morphological disorder and collagen fiber hyperplasia

**Fig. 3:**
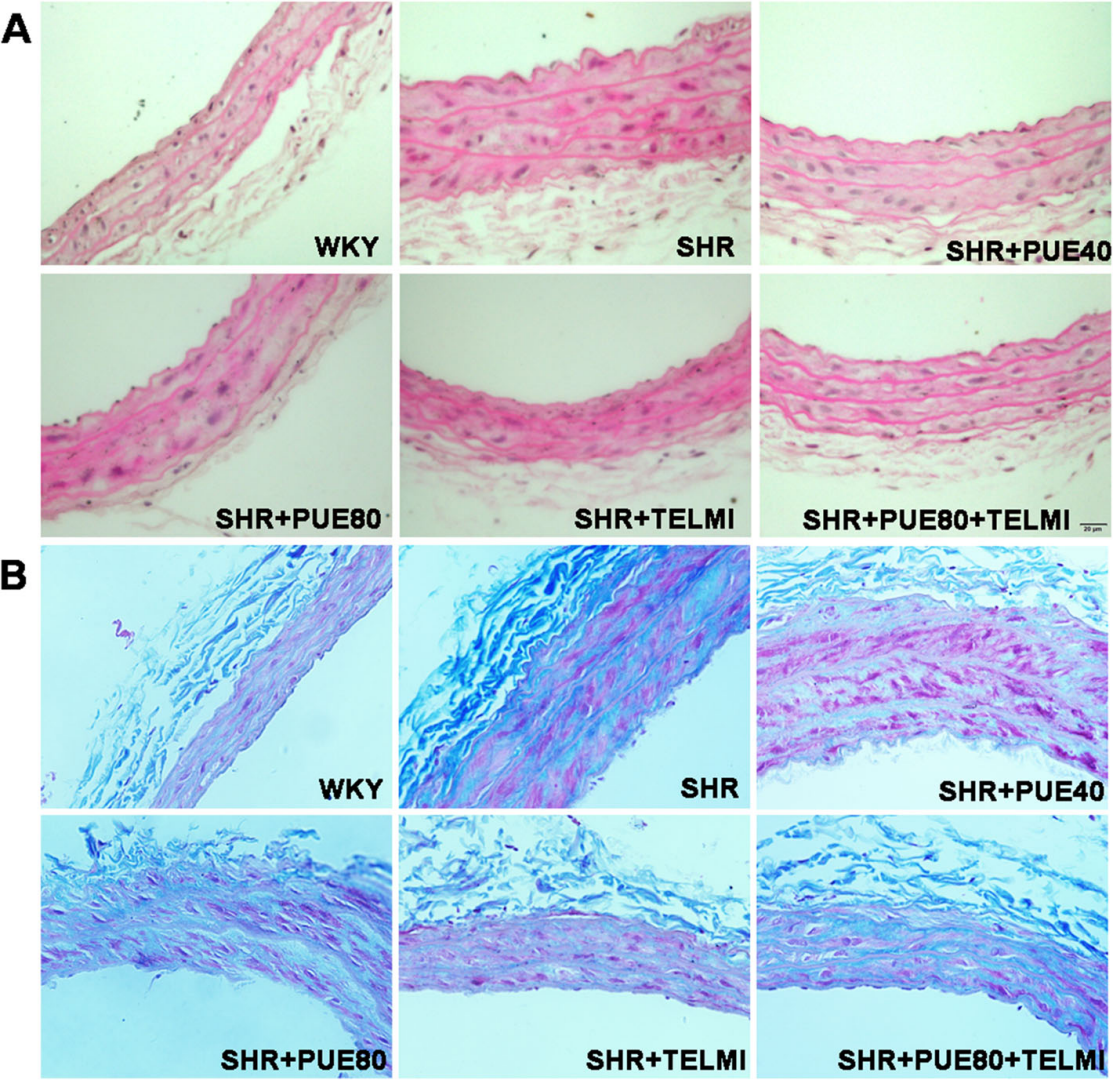
Effect of PUE on the morphological structure of common carotid artery. **A** Representative H&E-stained histological images of CCA in different groups; thickness of CCA of SHR group was significantly larger, and vascular smooth muscle cells were arranged in disorder, hypertrophy, and irregular in shape compared to WKY group; PUE led to a less damaged CCA. **B** Representative Masson-stained histological images of CCA in different groups. The collagen fibers stained blue-green of SHR group were obviously more than WKY group, and PUE could ameliorate CCA collagen fiber hyperplasia (Scale bar: 100 μm)

To observe the morphological abnormalities of CCA in each group, we choose HE and Masson stain. The results showed as in Fig. 3A, compared with WKY group, CCA thickness of SHR group was larger, and vascular smooth muscle cells were arranged in disorder, hypertrophy, and irregular shape, PUE led to a less damaged CCA. Masson stain was helpful to assess the degree of fibrosis, and the results were shown in Fig. 3B, compared with WKY group, the normal red muscle fibers of CCA in SHR group were covered by increased blue-green collagen fibers. After PUE intervention, the layer of smooth muscle became thinner, the degree of collagen fiber hyperplasia was reduced.

### PUE ameliorated NE-response of SHR rats

**Fig. 4:**
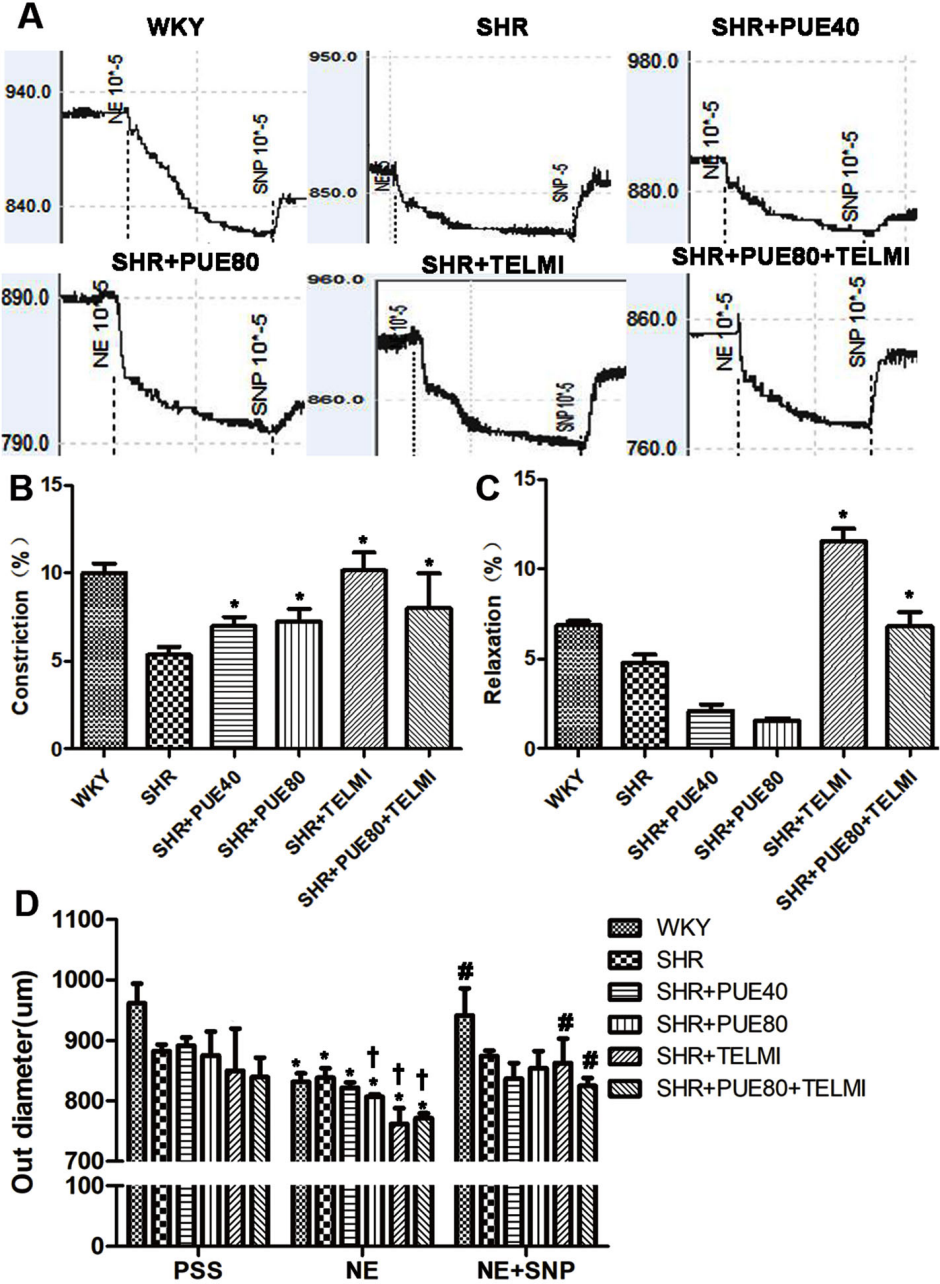
Representative functional response of common carotid artery at *P* = 60 mmHg. **A** Representative original tracings of functional curves induced by NE and SNP. **B** The histogram analysis of percentage of NE-induced contraction extent, * *p* < 0.05 vs SHR. **C** The histogram analysis of percentage of SNP-induced relaxation extent, * *p* < 0.05 vs SHR. **D** The histogram analysis of out diameter changes in different groups by NE and SNP, data in PSS is taken as the control. * *p* < 0.05 vs the same group in PSS, † *p* < 0.05 vs SHR group in NE, # *p* < 0.05 vs the same group in NE. (One-way ANOVA with Bonferroni post-hoc test)

NE is a VSMC-dependent vasoconstrictor mainly acting through the alpha1-adrenoceptors, it was used to test smooth muscle contractility; and SNP is an endothelial cell-independent vasodilator, it was used to test endothelial-independent smooth relaxation. The vasoconstriction reaction trend to NE was similar in all six groups, arteries from all six groups retained smooth muscle and endothelial function and exhibited a similar partial tone and vasoactive capacity following mechanical testing (Fig. 4A). Following attempted stimulation with NE, there was a near full dilatation back to baseline in response to SNP. NE-response of SHR group was obviously impaired compared to WKY group, and PUE led a less impaired response. As shown in Fig. 4B, NE-induced constriction of PUE-administrated groups was obviously bigger than SHR group. In Fig. 4C, SNP-induced relaxation was bigger just in the two TELMI groups, it showed TELMI significantly enhanced endothelial-independent smooth vasodilation. There was no significant SNP-induced vasodilation between PUE-administrated group and SHR group. Consistent with the results, the outer diameter just showed the same change in Fig. 4D. All these results suggested that PUE could ameliorate smooth muscle contractility property damaged by high blood pressure.

### PUE partly inhibited NE-induced passive vasoconstriction

**Fig. 5:**
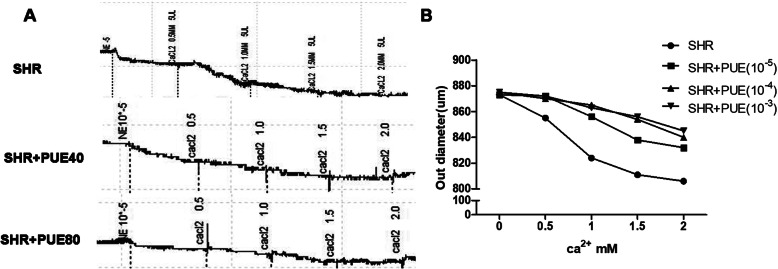
PUE inhibited NE-induced passive vasoconstriction. **A** Representative original tracings of NE- reactivity curves of SHR rats fed with PUE, PUE could obviously reduce NE-induced passive vasoconstriction as the calcium concentration increasing. **B** The outer diameter of carotid rings of SHR rats incubated with various concentrations of PUE, 10^− 3^ mol/L PUE could inhibit the outer diameter decreasing induced by increasing Ca^2+^

Passive mechanical characterization is critical for determining the effects of changes in the matrix composition of large elastic arteries due to development, aging, disease or injury and the subsequent effects on cardiac and cardiovascular function. In our study, NE was given to stimulate the isolated vascular ring to test CCA passive mechanical characterization of SHR group and SHR + PUE40/80 groups. As shown in Fig. 5A, long-term administration of PUE could reduce passive NE-induced vasoconstriction as calcium concentration increasing. In Fig. 5B, when CCA rings of SHR group were incubated with different concentrations of PUE, the results suggested that 10^− 3^ M PUE could obviously inhibit the decreasing of CCA outer diameters.

### The mechanical properties of carotid artery were closely related to TRPCs

**Fig. 6:**
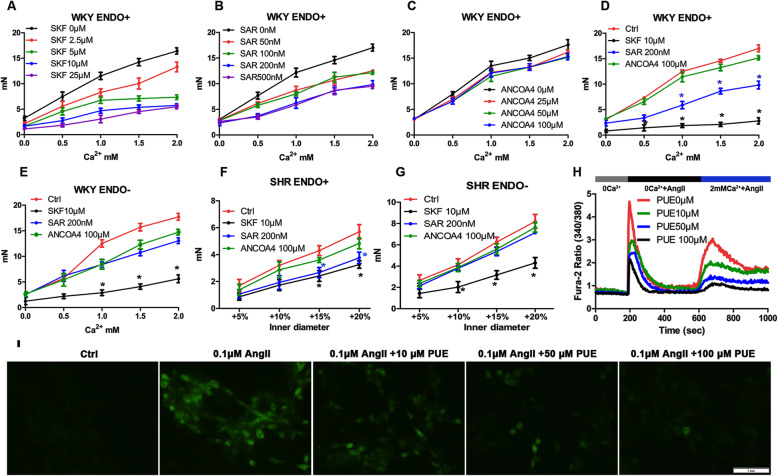
Ca^2+^-induced pressure changes were associated with TRPCs channel. **A**, **B**, **C**, **D** the pressure- Ca^2+^ response curves of CCA rings of WKY rats with intact endothelium, 10 μM SKF and 200 nM SAR could obviously decrease CCA pressure with endothelium compared to the control; 100 μM ANCOA4 has no effect on CCA pressure. **E** The pressure-Ca^2+^ response of CCA rings in WKY rats without endothelium, SKF could obviously decrease CCA pressure without endothelium compared to the control. **F**, **G** The pressure-inner diameter curves of CCA of SHR rats incubated with SKF, SAR or ANCOA4 with intact endothelium or without endothelium. **H** Time course of [Ca2+]_i_ changes as seen in Fura2-loaded A7R5 cells, PUE were incubated for 24 h before experiment, 0.1 μM AngII was used in the experiment. **I** Fluorescent dye Fluo-3/AM staining of A7R5 cells, Scale bar: 1 mm. SKF: SKF96365, pan-TRPCs inhibitor; SAR: SAR7334, specific TRPC6 inhibitor; ANCOA4:Orai1 inhibitor, * *p* < 0.05 vs Ctrl. (Two-way ANOVA with Bonferroni post-hoc test)

Physiological as well as maladaptive restructuring processes are associated with altered Ca^2+^ homeostasis in both vascular endothelium and smooth muscle. Both TRPC and Orai channels have been recently suggested to control phenotype of vascular cells and therefore to represent attractive novel targets for pharmacological prevention of vascular remodeling and aging [21, 22]. In order to further explore how PUE affects the biomechanical properties of CCA, we first used some Calcium ion channel inhibitors to gain further insight into the potential protective molecular mechanisms of PUE. We found 10 μM SKF could obviously decrease CCA pressure with endothelium compared to the control, and the effect of 10 μM SKF just equal to 25 μM in Fig. 6A. As shown in Fig. 6B, 200 nM SAR could also obviously decrease CCA pressure with endothelium. Figure 6C showed that CCA pressure with endothelium has little discernible change although ANCOA4 concentrations varied. So, we choose 10 μM SKF, 200 nM SAR and 100 μM ANCOA4 in the following experiment. As shown in Fig. 6D, pan-TRPCs inhibitor SKF and specific TRPC6 inhibitor SAR could obviously decrease pressure of WKY rats with intact endothelium, it illustrated some TRPCs channels played pivotal role in maintenance of mechanical properties. In Fig. 6E, the effect of SKF was still obvious in WKY rats without endothelium, the effect of SAR was not so obvious, so SAR might play roles through vascular endothelium. As shown in Fig. 6F, SKF and SAR could significantly decrease the increasing pressure level of SHR while the inner diameter increasing with intact endothelium, but without endothelium just SKF had obvious effect on decreasing the pressure in Fig. 6G, this further confirmed that some TRPC channels took part in CCA mechanics and remodeling of SHR rats. As shown in Fig. 6H, Ca^2+^-imaging experiment showed that PUE can reduce calcium influx induced by AngII in A7R5 cells. in Fig. 6I, PUE can obviously decrease cytoplasmic Ca^2+^ concentration of A7R5 cells induced by AngII.

### PUE ameliorated vascular mechanical properties through inhibiting TRPCs channel activities

**Fig. 7:**
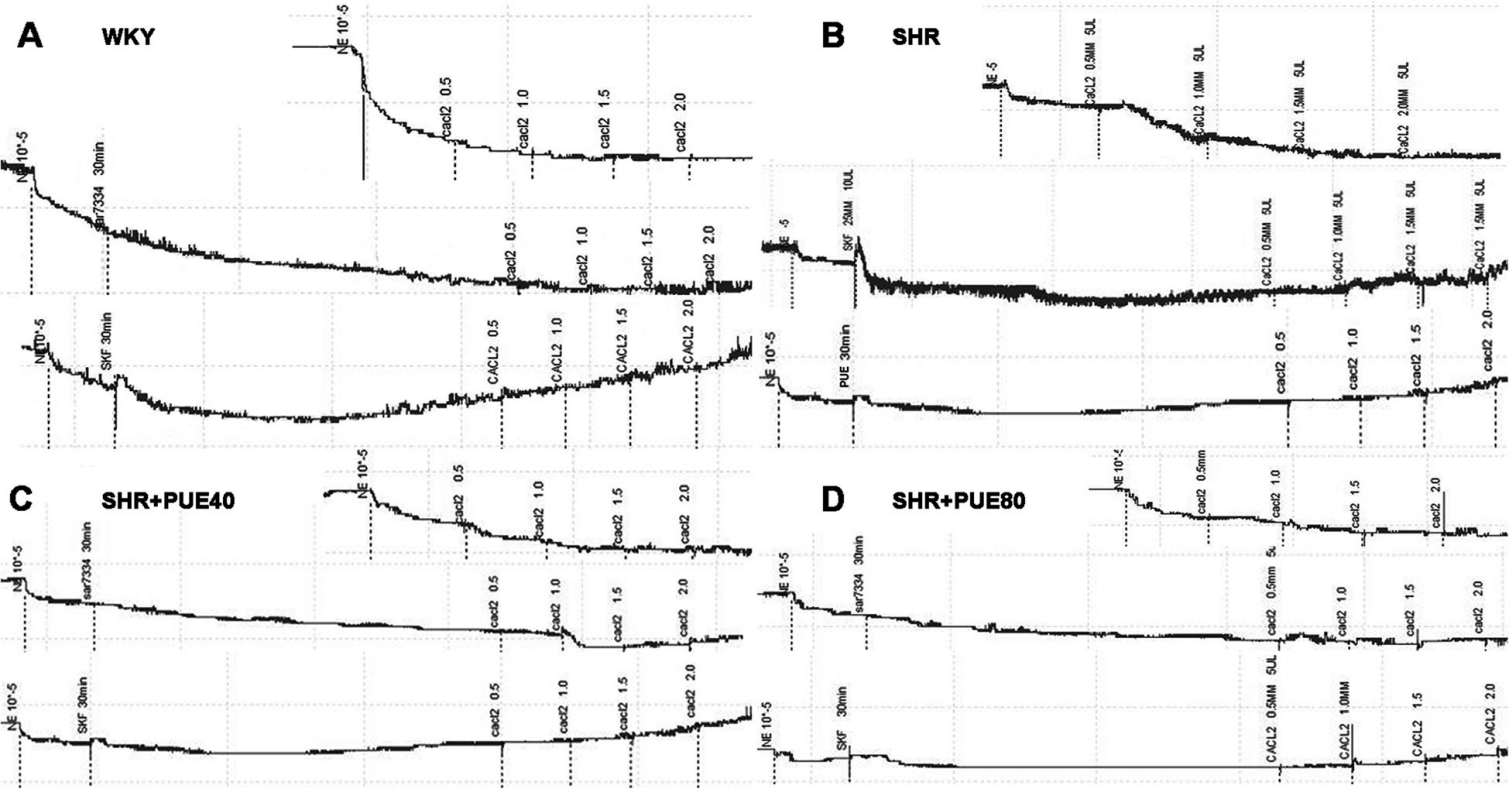
PUE ameliorated Ca^2+^-induced constriction with the similar role as SKF. **A** Representative original tracings of NE-reactivity curves of WKY rats, SKF could obviously inhibit the decrease of outer diameter of carotid rings induced by increasing extracellular Ca^2+^. **B** Representative original tracings of NE-reactivity curves of SHR rats incubated with 10^− 3^ M PUE, PUE played the similar role as SKF. **C** Representative original tracings of NE-reactivity curves of SHR fed with 40 mg/kg/d of PUE. **D** Representative original tracings of NE-reactivity curves of SHR fed with 80 mg/kg/d of PUE; SKF has no obvious effect on inhibition the outer diameter decrease induced by increasing extracellular Ca^2+^

Initiation and regulation of vascular smooth muscle contraction involves a unique repertoire of Ca^2+^ entry mechanisms [23]. Importantly, the underlying Ca^2+^ entry channels comprise both voltage-gated, highly selective channels as well as nonselective cation channels. As shown in Fig. 7A, SKF could partly inhibit the decreasing of outer diameter induced by NE in WKY rats, the effect of SAR was not so obvious as SKF. In order to further explore the protective effect of PUE, we incubated CCA rings of SHR groups with SKF or PUE for 30 min differently, as shown in Fig. 7B, SKF could significantly inhibit the decreasing of outer diameter, and PUE could take the similar effect as SKF. As shown in Fig. 7C and D, 12 weeks administration of PUE (40 mg/kg/d or 80 mg/kg/d) could reduce the passive NE-induced vasoconstriction, SKF incubated for 30 min had no obvious effect on reducing outer diameter, the reasonable explanation was that long term administration of PUE had decreased the expression of TRPCs channel, or PUE had directly inhibited some TRPCs channels, including TRPC6.

## Discussion

In the present study, we showed that PUE could significantly decrease SBP and DBP, obviously decrease thickness and inner diameter of CCA, the explanation is the apparently stiffer behaviors of the arteries in terms of a phenotypic modulation of VSMCs from more of a contractile to more of a synthetic phenotype. Arteries from all six groups retained smooth muscle and endothelial function and exhibited a similar partial tone and vasoactive capacity following mechanical test. Vascular agonists and Ca^2+^ channel antagonists were applied outside of the pressurized artery(extraluminally), which is more representative of vasodilatory mediators released from surrounding tissue and/or parasympathetic or sympathetic innervation of vascular smooth muscle cells, allowing interaction with endothelium indirectly. This is a more physiological method of measuring the vascular endothelial independent reaction of vascular smooth muscle cells, not the circulating or endothelium-derived vasodilators.

PUE affects multiple signaling molecules including the inhibition of oxidative stress, and the NFκB and MAPK activation in vasculature and heart, which may contribute to its suppression of hypertension-induced cardiac hypertrophy and remodeling and improvement of vascular function [24–26]. PUE produced vasodilation via an endothelium-dependent mechanism involving NO [27–31] and an endothelium-independent pathway mediated by the opening of K^+^ channels in arteries [15, 32–34]. Vascular smooth muscle contraction is initiated by both calcium-dependent and -independent mechanisms, the structure and functional abnormal of calcium channels may lead to the occurrence and development of hypertension [35, 36], but the effect of PUE on Ca^2+^ have no consensus yet.

As shown in our studies, the reactivity of Ca^2+^-induced vasoconstriction is impaired in SHR rats, PUE can rescue this condition. Calcium imaging of A7R5 cells also illustrated that PUE could decrease the Ang II-induced Ca^2+^ influx. But the regulation role of PUE against Ca^2+^ influx still remains controversial, some reports confirmed that long-term PUE treatment enhances Ca^2+^ reuptake and Ca^2+^ content via upregulation of SERCA2a of murine embryonic stem cell-derived cardiomyocytes [17], and PUE restrained Ca^2+^ influx and reduced [Ca^2+^]_i_ in daunorubicin-incubated H9c2 cells [18], another study reported PUE induced the [Ca^2+^]_i_ increase by evoking phospholipase C-independent Ca^2+^ release from the endoplasmic reticulum and other unknown stores in MDCK and renal tubular cells [19]. Our experiment supplements the evidence that PUE can decrease calcium influx induced by Ang II.

Ca^2+^ entry from voltage-gated Ca^2+^ channels are mainly responsible for excitation-contraction coupling, non-voltage-dependent Ca^2+^ entry channels such as Orai, STIM, and TRPCs functions have been linked to physiological downstream pathways that are essential for vascular remodeling [37–39]. Membrane proteins of the TRPC family have been recognized as pivotal elements of Ca^2+^ transcription coupling [40] and demonstrated to control phenotype transitions, proliferation, and differentiation in smooth muscle and endothelium [41]. Ca^2+^ signaling mediated by TRPC channels determines initiation and progress of vascular remodeling via signaling processes that govern transcriptional programs and phenotype transitions [42–44]. Similarly to the endothelium, all members, except TRPC2, of all the TRPC subfamily have been detected in human vasculature by PCR, Western Blot, and/or immunofluorescence analysis [45, 46]. Most studies focusing on TRPCs function and distribution in terms of VSMC remodeling revealed an increased expression of these ion channels in proliferating VSMC of diverse disease models [47–49].

As shown in our studies, in SHR rats, SKF96365 and SAR7334 could decrease the increasing level of pressure with intact endothelium, just SKF96365 had obvious change on decreasing the CCA pressure without endothelium, this further confirmed that some TRPC channels took part in the mechanics and remodeling of CCA of SHR rats via VSMC. As shown in Fig. 7B, SKF could significantly inhibit the decreasing of outer diameter of SHR rats, PUE could take the similar effect as SKF. While 12 weeks administration of PUE (40 mg/kg/d or 80 mg/kg/d) could reduce the passive NE-induced vasoconstriction, and SKF had no effect in these two PUE administration groups, the reasonable explanation was that PUE ameliorated Ca^2+^-induced constriction with the similar role as SKF, long term administration of PUE has decreased the expression of some TRPCs channel, or PUE has directly inhibited some TRPCs channels including TRPC6, but which specific TRPC channel PUE acts on remains to be further explored.

**Fig. 8:**
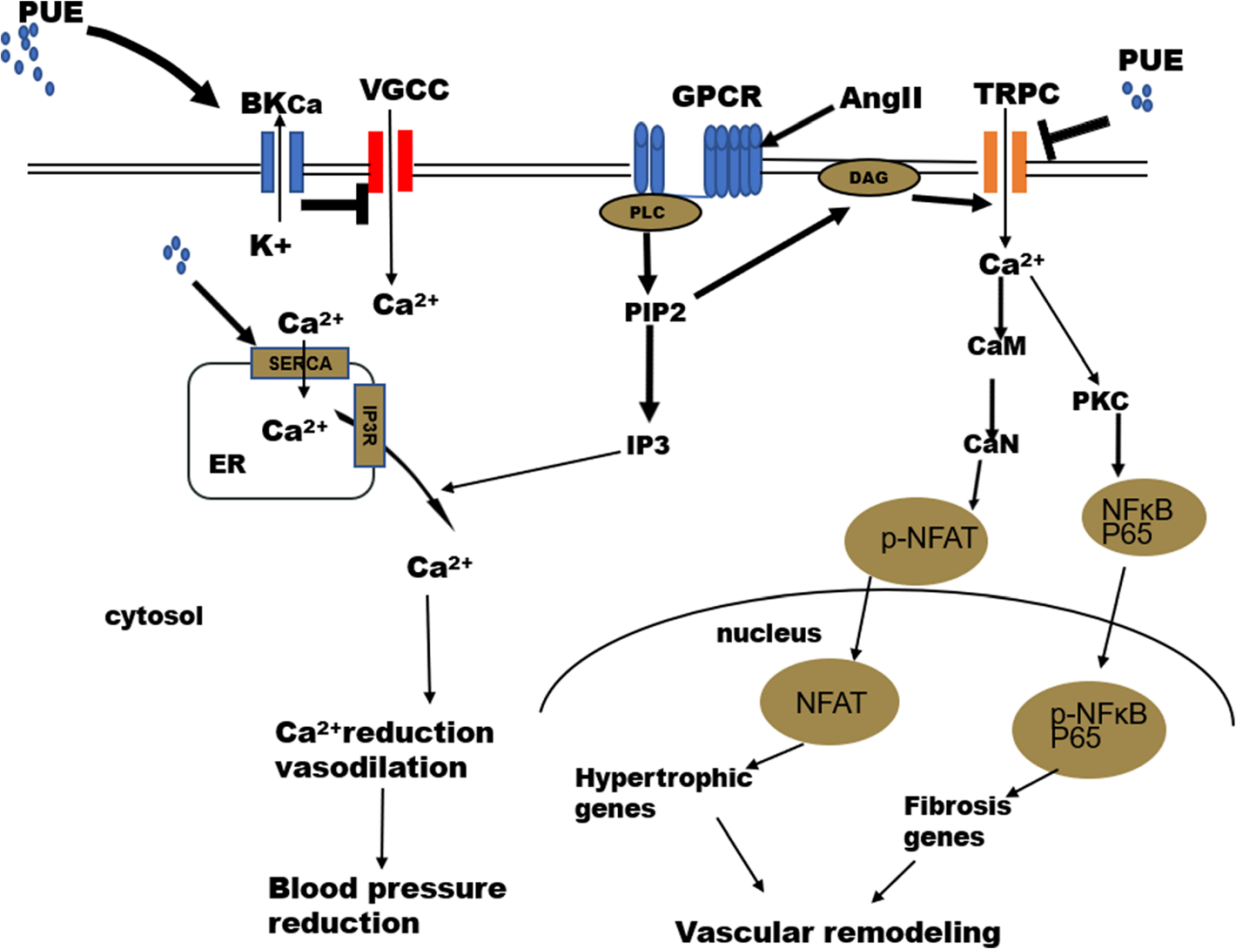
Underlying effect and possible mechanism of PUE on blood pressure and vascular remodeling involving Ca^2+^ in hypertension. The mechanism by which PUE reduced blood pressure was mainly related to BK_Ca_ activation, VGCC inactivation and SERCA activation. The mechanism by which PUE ameliorated vascular remodeling was mainly related to TRPCs inactivation, regulating the Ca^2+^-CaN-NFAT pathway and Ca^2+^-PKC-NFκB pathway. BK_Ca_: large-conductance voltage- and Ca^2+^-activated potassium channel; VGCC: Voltage gated calcium channels; SERCA: sarco endoplasmic reticulum Ca^2+^-ATPase; CaM: Calmodulin; CaN: Calcineurin; PKC: protein kinase C; GPCR: G protein coupled receptor; PLC: phospholipase C

Here, just to summarize the underlying effect and possible mechanism of PUE on blood pressure and vascular remodeling involving Ca^2+^ in hypertension. As shown in Fig. 8, the mechanism by which PUE reduced blood pressure was mainly related to BK_Ca_ activation. PUE activated BKCa channels, then leads to hyperpolarization of the cell membrane, which causes deactivation of voltage-dependent calcium channels and vasodilation [15, 34], SERCA activation caused by PUE can also lead to vasodilation [17, 18]. We postulated that long time administration of PUE ameliorated vascular remodeling was mainly related to TRPCs inactivation, which reduced calcium overload induced by angiotensin II, Calcium influx through TRPC channels were reduced, which regulating the Ca^2+^-CaN-NFAT pathway [39, 46–49] and Ca^2+^-PKC-NFκB pathway [24, 26], thus influence the vascular remodeling.

All the above-mentioned findings indicate that PUE represents a potential antihypertensive agent, it reduces the blood pressure of SHR rats. PUE can also set off a chain of events and ultimately improves the mechanical properties and remodelings of CCA via blocking TRPCs. But in our study, the specific molecular target of PUE acts on and how PUE affect TRPCs should be assessed in follow-up work. Our findings raise some interesting additional questions about PUE that need to be answered.

## Supplementary Information

**Additional file 1.**

## Data Availability

All data is contained within the manuscript.
