# Supplementary figures and images for: Ameliorated biomechanical properties of carotid arteries by puerarin in spontaneously hypertensive rats
Source: BMC Complement Med Ther. 2021 Jun 22;21:173. doi: 10.1186/s12906-021-03345-8 (PMC8216761; doi:10.1186/s12906-021-03345-8)

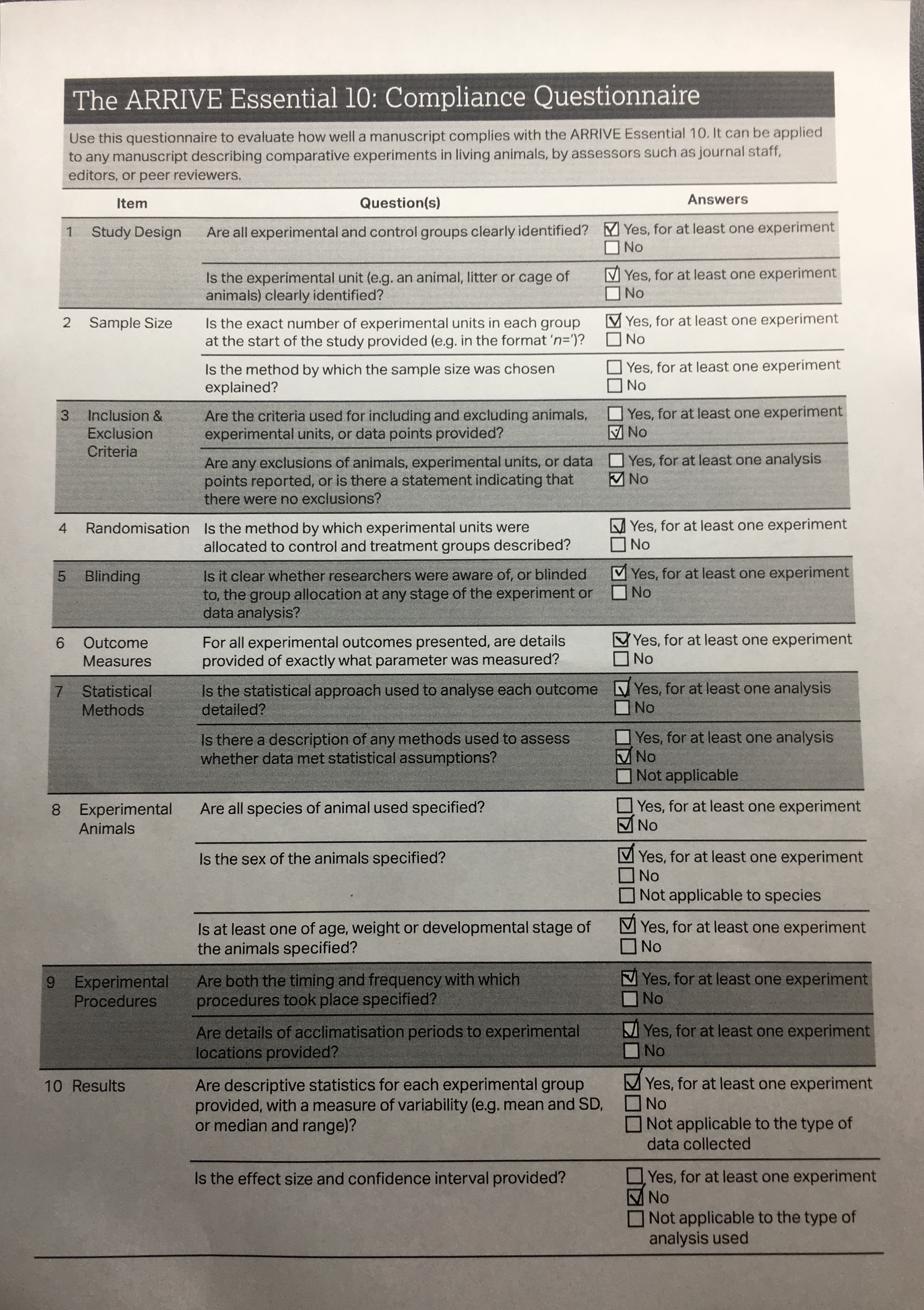

Supplement: Supplementary file 1 — Additional file 1. [file 12906_2021_3345_MOESM1_ESM.jpg]
